# Automated closed–loop FiO_2_ titration increases the percentage of time spent in optimal zones of oxygen saturation in pediatric patients–A randomized crossover clinical trial

**DOI:** 10.3389/fmed.2022.969218

**Published:** 2022-08-25

**Authors:** Ekin Soydan, Gokhan Ceylan, Sevgi Topal, Pinar Hepduman, Gulhan Atakul, Mustafa Colak, Ozlem Sandal, Ferhat Sari, Utku Karaarslan, Dominik Novotni, Marcus J. Schultz, Hasan Agin

**Affiliations:** ^1^Department of Pediatric Intensive Care Unit, Dr. Behcet Uz Children's Disease and Surgery Training and Research Hospital, Health Sciences University, Izmir, Turkey; ^2^Department of Medical Research, Hamilton Medical AG, Bonaduz, Switzerland; ^3^Department of Intensive Care, Amsterdam UMC, Location “Academic Medical Center”, Amsterdam, Netherlands

**Keywords:** intensive care, pediatric intensive care, mechanical ventilation, Adaptive support ventilation, ASV, closed loop ventilation, automated ventilation, FiO_2_ controller

## Abstract

**Introduction:**

We aimed to compare automated ventilation with closed–loop control of the fraction of inspired oxygen (FiO_2_) to automated ventilation with manual titrations of the FiO_2_ with respect to time spent in predefined pulse oximetry (SpO_2_) zones in pediatric critically ill patients.

**Methods:**

This was a randomized crossover clinical trial comparing Adaptive Support Ventilation (ASV) 1.1 with use of a closed–loop FiO_2_ system vs. ASV 1.1 with manual FiO_2_ titrations. The primary endpoint was the percentage of time spent in optimal SpO_2_ zones. Secondary endpoints included the percentage of time spent in acceptable, suboptimal and unacceptable SpO_2_ zones, and the total number of FiO_2_ changes per patient.

**Results:**

We included 30 children with a median age of 21 (11–48) months; 12 (40%) children had pediatric ARDS. The percentage of time spent in optimal SpO_2_ zones increased with use of the closed–loop FiO_2_ controller vs. manual oxygen control [96.1 (93.7–98.6) vs. 78.4 (51.3–94.8); *P* < 0.001]. The percentage of time spent in acceptable, suboptimal and unacceptable zones decreased. Findings were similar with the use of closed-loop FiO_2_ controller compared to manual titration in patients with ARDS [95.9 (81.6–98.8) vs. 78 (49.5–94.8) %; *P* = 0.027]. The total number of closed-loop FiO_2_ changes per patient was 52 (11.8–67), vs. the number of manual changes 1 (0–2), (*P* < 0.001).

**Conclusion:**

In this randomized crossover trial in pediatric critically ill patients under invasive ventilation with ASV, use of a closed–loop control of FiO_2_ titration increased the percentage of time spent within in optimal SpO_2_ zones, and increased the total number of FiO_2_ changes per patient.

**Clinical trial registration:**

ClinicalTrials.gov, identifier: NCT04568642.

## Introduction

Critically ill invasively ventilated children frequently need supplementary oxygen, which is provided in humidified warmed air, either *via* a stand-alone gas blender or *via* a blender inside the ventilator. Both hypoxemia and hyperoxemia should be adequately responded to, either by increasing or lowering the fraction of inspired oxygen (FiO_2_), as deteriorations in pulse oximetry (SpO_2_) readings are associated with worse outcomes, including a higher mortality (1), development of retinopathy, chronic lung disease, and brain injury (2).

The Pediatric Acute Lung Injury Consensus Conference (PALICC) guidelines suggest targeting “safe” SpO_2_ targets at the lowest possible FiO_2_. Obviously, this comes with challenges. First, a too low SpO_2_ increases the risk of mortality (3). Second, with use of the lowest possible FiO_2_, children may develop dangerous hypoxemia much easier. Last but not least, maintaining SpO_2_ in predefined target ranges in critically ill children is difficult due to frequent fluctuations in saturation caused by their respiratory instability. The scarcity of doctors and nurses skilled in titrating FiO_2_ or inadequate resources to adhere to a tight manual FiO_2_ regimen exacerbates these issues.

Automated, or closed–loop FiO_2_ control could prevent hyperoxemia and hypoxemia, incorrect use of oxygen, and reduce the workloads of intensive care unit staff. A recent meta-analysis suggest that use of automated FiO_2_ titrations are associated with improvement in terms of the time spent in target SpO_2_ ranges, reduces periods of hyperoxia and severe hypoxia on positive pressure respiratory support in preterm infants (4). It is uncertain whether this the use of closed–loop FiO_2_ control has similar effects in pediatric patients. Therefore, we evaluated the performance of a closed–loop FiO_2_ titration system in pediatric patients under Adaptive Support Ventilation. We hypothesized that oxygenation would be safer and more efficient with the use of a closed–loop FiO_2_ titration system.

## Methods

### Study design and ethics

This was a single center randomized crossover clinical trial, performed in the Dr. Behcet Uz Children's Research and Training Hospital, Izmir, Turkey. The study was conducted in accordance with the Declaration of Helsinki, the study protocol was approved by the local Institutional Review Board, and the study was registered at clinicaltrials.gov (NCT04568642).

### Patient selection

Patients were eligible for participation if: (1) aged between 1 month and 18 years; (2) with an ideal body weight of 7 kg; (3) receiving invasive ventilation with an FiO_2_ ≥ 25%; and after having received written informed consent from the legal representative. Patients were excluded if hemodynamic instable, or when it was expected that they would not stay stable in the next 5 h, or when there were air leaks around the endotracheal tube ≥40%. Patients could also not participate if the legal representatives had not given written informed consent. We also excluded patients with congenital or acquired hemoglobinopathies affecting SpO_2_ readings.

### Collected data

Demographic data collected using a case report form (CRF) were age (months), gender, height (cm), ideal body weight for the measured height (kg), pediatric index of mortality (PIM) score, admission diagnosis, and lung physiology during the study. Additionally, arterial blood gas (ABG) data were recorded to the CRF.

Breath–by–breath ventilation parameters collected using a MemoryBox (Hamilton Medical AG, Bonaduz, Switzerland) included FiO_2_ and SpO_2_, positive end expiratory pressure (PEEP), mean airway pressure (MAP), minute ventilation volume (MinVent), respiratory rate (RR), end-tidal CO_2_ (EtCO_2_).

### Measurement and calculation

A single-use flow sensor (Hamilton Medical AG, Bonaduz, Switzerland) was placed between the endotracheal tube and the Y-piece (8) to measure the airway pressures and flows, while volume was obtained by integrating the flow signal. CO_2_ measurements were obtained using a mainstream CO_2_ sensor (Capnostat5, Philips GmbH, Germany) together with the corresponding adult/pediatric airway adapter that has a dead-space volume of 5 ml (5). Ventilation parameters were measured at each breath. Patients' SpO_2_ was monitored by means of a Masimo Set sensor attached to their finger (Masimo RD; Masimo Corp., Irvine, CA, USA) to provide the signal used by the closed-loop controller.

### Ventilation protocol

Both spontaneously breathing and passive patients were enrolled in the study. They were intubated with an appropriately sized and inflated cuffed tube and ventilated in a semi-recumbent position with a Hamilton-S1 ventilator. If required, patients were sedated and if necessary paralyzed according to the local protocol for sedation and using Comfort behavior scale (6). The IntelliCuff device (Hamilton Medical AG, Bonaduz, Switzerland) was used to target a cuff pressure slightly lower than the patients' peak inspiratory pressure (PIP) level. In auto mode, relative cuff pressure was adjusted 2–5 cm H2O lower than PIP to ensure minimal leak around the ETT with minimal administered cuff pressure. However, according to pediatric ventilation guidelines, the maximum cuff pressure was limited to 20 cm H2O (7). Active humidification with the Hamilton-H900 (Hamilton Medical AG, Bonaduz, Switzerland) was used as required.

**Figure 1:**
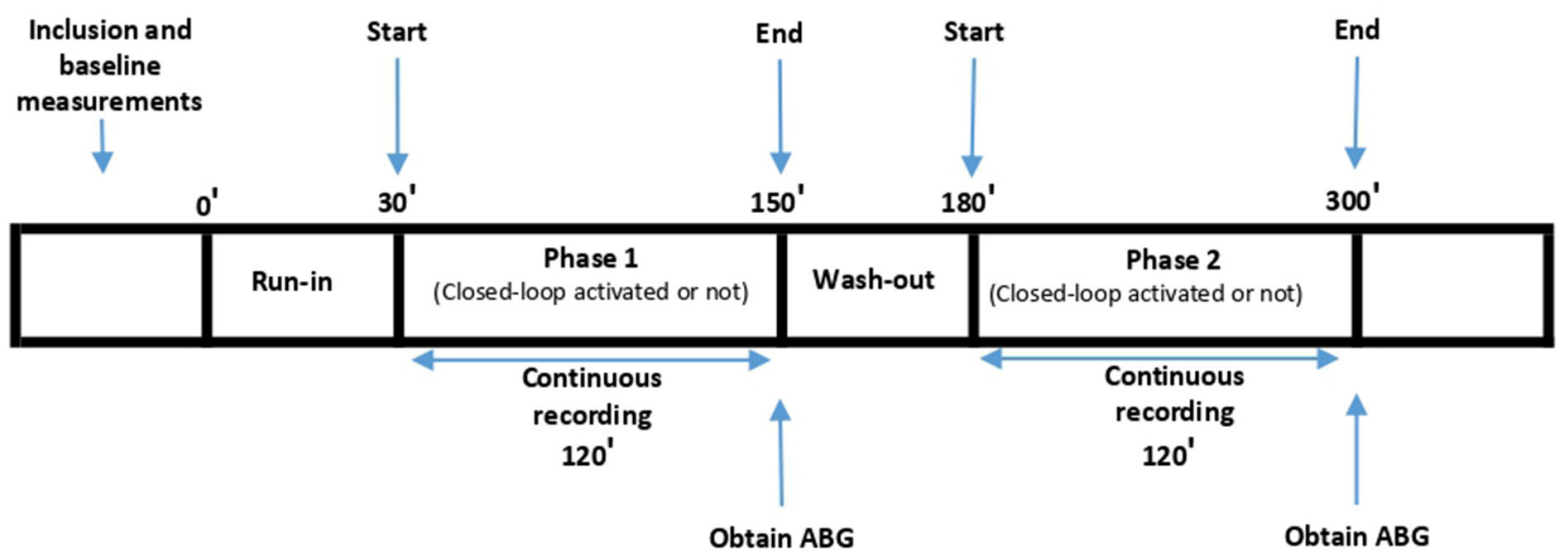
Study Protocol. Run-in: Patient was ventilated with the same mode as Phase 1, excluded from data analysis; Wash-out: Patient was ventilated with the same mode as Phase 2, excluded from data analysis.

Ventilation was started with standard Adaptive Support Ventilation (ASV) 1.1 settings. ASV 1.1 is a closed-loop ventilation mode in which the clinician first enters the height of the patient to calculate ideal body weight, then determines the percentage of MinVent to be multiplied by the patient's ideal body weight. Then, using the Otis and Mead equations, this MinVent is distributed algorithmically between RR and tidal volume (VT) respecting expiratory time constant (RCexp) of the patient in order to accomplish minimum work of breath and minimum applied pressure. The attending physician subsequently checked the breathing parameters according to the study protocol and wrote them on the CRF while he began collecting data with the MemoryBox in mixed mode. Afterwards, the clinician started the first phase of the study continuing ventilation with standard ASV 1.1 with with only the FiO2 controller activated, the PEEP and minute volume controller deactivated, according to randomization. After 2.5 h of recording in the first phase, the clinician switched the patient to the alternate mode, according to the randomization order. If the patient was ventilated without the FiO_2_ controller activated in the first phase, the Intellivent FiO_2_ controller was activated in the second phase. The patient remained in the second phase for 2.5 h as well. The first 0.5 h of the first phase were considered as a run-in phase and the first 0.5 h of the second phase were considered as a wash-out phase. Therefore, the first 0.5 h of each phase were excluded from data analysis. For each patient we collected data for 120 min with the FiO_2_ controller activated and for 120 min with the FiO_2_ controller deactivated, and these time periods were compared with regard to the primary and secondary endpoints (Figure 1). The same values for both MinVent and PEEP were maintained during the two phases. Intellivent FiO_2_ closed-loop controller is a rule based, proportional integral controller described in Supplementary Table 1. Manual titration protocol is described in the same file, too.

### Endpoints

The primary endpoint of the study was the percentage of time spent in a predefined optimal SpO_2_ range over the 2-h observation periods. Secondary endpoints were the percentage of time spent in the predefined acceptable, suboptimal, and unacceptable SpO_2_ ranges, as well as the number of FiO_2_ adjustments (manual or performed automatically by the closed-loop controller).

### Definitions

The definitions for optimal, acceptable, suboptimal, and unacceptable SpO_2_ ranges are provided in Table 1. We had different predefined cut-offs for the patients at higher and lower PEEP based on PALICC and PEMVEC guidelines (7, 8).

**Table 1:** SpO2 (Peripheral oxygen saturation) ranges.

**Group**	**Unacceptably low**	**Suboptimally low**	**Acceptably low**	**Optimal**	**Acceptably high**	**Suboptimally high**
Lower PEEP (<10 cmH2O)	<85%	≥85 and <90%	≥90 and <93%	≥93 and ≤97%; >97% if FiO2 = 0.21	>97 for ≤ 60 s	>97 for >60 s
HigherPEEP (≥10 cmH2O)	<80%	≥80 and <85%	≥85 and <88%	≥88 and ≤92%; >92% if FiO2 = 0.21	>92 for ≤ 60 s	>92 for >60 s

PEEP, positive end expiratory pressure; FiO2, Fraction of inspired oxygen.

### Power calculation and statistical analysis

The sample size was calculated by means of a pilot study in seven patients. In those seven patients the mean difference between the two phases was 12 ± 19%, median time spent in the optimum range were 86% [55–99 (IQR)] vs. 67% [50–81 (IQR)]. Based on this pilot data, G^*^Power computed that we needed 30 participants to detect an effect size of Cohen's d = 0.64 with 95% power (α = 0.05, one-tailed) in a Wilcoxon signed-rank test (9). Shapiro-Wilk, skewness and kurtosis normality tests were used to check the distribution of data. Continuous data were expressed in terms of either mean and standard deviation or median and interquartile range (IQR). We used a paired samples *t*–test to compare normally distributed data, and the Wilcoxon test when data were not normally distributed. The Wilcoxon signed-rank test was used for the comparison between the percentage of time spent in the target SpO2 range with manual FiO_2_ control and the percentage with closed-loop FiO_2_ control, and *p*-values of <0.05 were considered to be statistically significant for all comparisons. Statistical testing was carried out with the XLSTAT statistical package (version 2016).

## Results

### Patients

**Figure 2:**
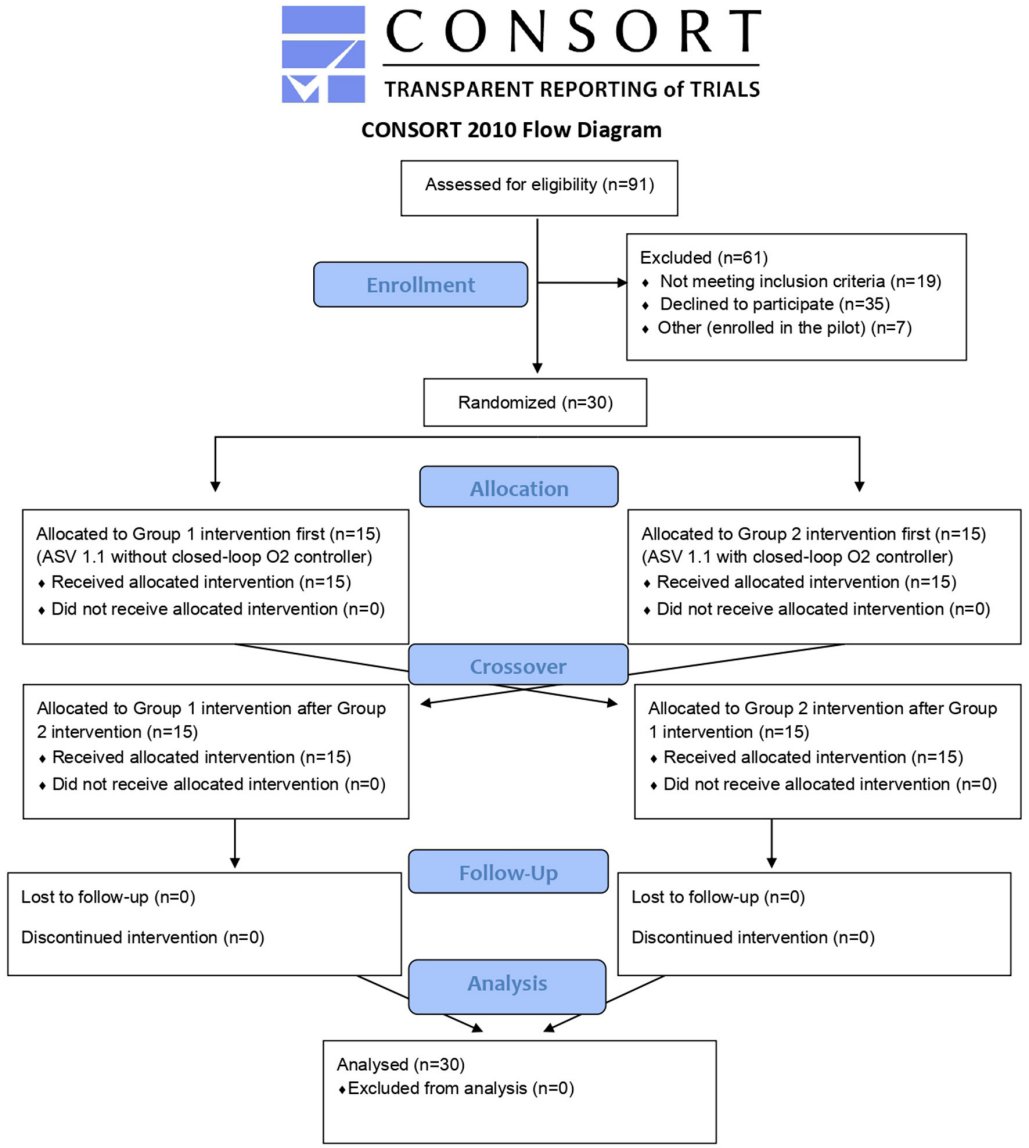
Consort 2010 flow diagram.

During the period from October 2020 and April 2021, investigators in the PICU of the Dr. Behcet Uz Children's Research and Training Hospital screened a total of 91 intubated and mechanically ventilated patients for inclusion. Of those, 19 were excluded due to meeting at least one of the exclusion criteria and informed consent was not given for a further 35 patients. Of the 37 included patients, the first seven were enrolled for the pilot study and the remaining 30 were enrolled in the present study (Figure 2). Their baseline characteristics, which include their oxygenation index (OI) (10), and pediatric index of mortality 3 (PIM3) scores (11), can be seen in Table 2.

**Table 2:** Baseline characteristics of the patients.

**Variables**	
Gender ratio (%f/%m)	40/60
Age (months)	21 (11–48)
Height (cm)	93.5 (71.8–111.5)
IBW (kg)	13.5 (8.8–19.8)
OI	3.9 (3–7)
PIM3	11.9 (5.9–21.2)
**Admission diagnosis (** ***n*** **, %)**	
**Respiratory** *A.pneumonia* *A.bronchiolitis* *LRTI* *Chylothorax* *Laryngotracheomalacia*	9, 30
**Neurologic** *SE* *Meningoencephalitis*	7, 23
**Renal/Metabolic** *RTA* *HUS* *DKA*	5, 17
**Cardiovascular** *VSD* *PDA* *AS*	3, 10
**HAI** *CLABSI*	2, 7
**Other** *Trauma* *Gastrointestinal surgery*	4, 13
**Lung physiology (** ***n*** **, %)**	
Restrictive	10, 33
Mixed	10, 33
Obstructive	5, 17
Normal	5, 17

Data are expressed in median and interquartile range (25th−75th) or percentage.

IBW, Ideal body weight; ABW, Actual body weight; OI, Oxygenation index, PIM3, Pediatric index of mortality 3, probability of death; A.pneumonia, Acute pneumonia; A.bronchiolitis, Acute bronchiolitis; LRTI, Lower respiratory tract infection; VSD, Ventricular septal defect; PDA, Patent ductus arteriosus; AS, Aortic stenosis; RTA, Renal tubular acidosis; HUS, Hemolytic uremic syndrome; DKA, Diabetic Keto Acidosis; SE, Status epilepticus; HAI, Healthcare-associated infections; CLABSI, Central line related blood stream infection.

Ten patients had restrictive lung disease, while five had obstructive and a further ten mixed lung disease. Five patients had a normal lung condition. Patient's respiratory mechanics and ventilation variables during the two study phases are shown in Table 3. Also, ABG parameters were presented in Table 4. Seven of the 30 patients met the criteria for mild pediatric acute respiratory distress syndrome (PARDS), four for moderate and one for severe PARDS. The way the study was designed meant there was no change to either MinVent or PEEP.

**Table 3:** Ventilation parameters during study phases.

**Ventilation variable**	**ASV + FiO_2_ controller (*n =* 30)**	**ASV + Manual FiO_2_ (*n =* 30)**	**p1**
MinVent (l/min)	2.9 (2.4–3.5)	2.9 (2.4–3.4)	0.642
VT/IBW (ml/kg)	7.5 (6.4–8.1)	7.4 (6.3–8.4)	0.19
RR (b/min)	29.3 (23.1–38.8)	29.5 (24.3–35.9)	0.257
PetCO_2_ (mmHg)	42 (33.4–50.3)	42.8 (33.7–54.1)	0.17
PIP (cmH2O)	20.2 (14–26.6)	20 (15.4–25.5)	0.719
PEEP (cmH2O)	5 (5–6.5)	5 (5–6.5)	1
FiO_2_ (%)	31.4 (25.1–44.1)	36.5 (30–49)	<0.001
FiO_2_ adjustment (n/2h)	52 (11.8–67)	1 (0–2)	<0.001
Ti (s)	0.7 (0.6–0.8)	0.7 (0.6–0.8)	0.234
Te (s)	1.3 (0.9–1.7)	1.4 (0.9- 1.7)	0.176
SpO_2_ (%)	96.2 (95.8–96.8)	97 (95.2–97.6)	0.245
SpO_2_ DOT (%)	96.4 (93.6–98.7)	78.4 (51.4–98)	<0.001
OI	3.1 (2.4–6)	3.5 (2.6–6.6)	<0.001
S/F	300.7 (210.8–382.2)	254.1 (189.2–302.4)	<0.001
TO_2_ (l/min)	0.3 (0.1–0.7)	0.5 (0.2–0.9)	<0.001

Data are expressed in median and interquartile range (25–75th) and compared between ASV+closed loop FiO_2_ controller and ASV+ Manuel FiO_2_ titration groups by Wilcoxon signed-rank test (p1).

ASV, Adaptive support ventilation; FiO2, fraction of inspired oxygen; VT, tidal volume expired; IBW, ideal body weight; RR, respiratory rate; PetCO_2_, end-tidal carbondioxide; PIP, peak inspiratory pressure; Ti, inspiratory time; Te, expiratory time; SpO_2_, peripheral oxygen saturation; SpO_2_DOT, peripheral oxygen saturation duration in optimal target; OI, oxygenation index; S/F, peripheral oxygen saturation to fraction of inspired oxygen ratio; TO2, therapeutic oxygen use.

**Table 4:** ABG parameters.

**ABG variable**	**ASV + Closed-loop FiO2 (*n =* 30)**	**ASV + Manual FiO2 (*n =* 30)**	**p1**
pH	7.36 (7.24–7.44)	7.36 (7.23–7.43)	0.13
PaO2 (mmHg)	97.5 (89.5–114.5)	102 (91.8–117)	0.936
PaCO2 (mmHg)	38.5 (32.8–48.3)	38 (32.7–46.5)	0.873

Data are expressed in median and interquartile range (25–75th) and compared between Closed-loop FiO2 and manual FiO2 group by Wilcoxon signed-rank test (p1).

ABG, Arterial blood gas; ASV, Adaptive support ventilation; PaO2, partial oxygen content; PaCO2, partial carbon dioxide content.

### Time spent in optimal SpO_2_ zones

Patients spent more time in the target SpO_2_ range when the FiO_2_ controller was activated. During the study phase with closed-loop FiO_2_ control, patients spent 96.1% (93.7–98.6 [IQR]) of their time in the optimal range compared to 78.4% [51.3–94.8 (IQR)] of their time in the optimal range when FiO_2_ was controlled manually (*p* < 0.001).

### Time spent in acceptable, suboptimal, and unacceptable SpO_2_ zones

**Figure 3:**
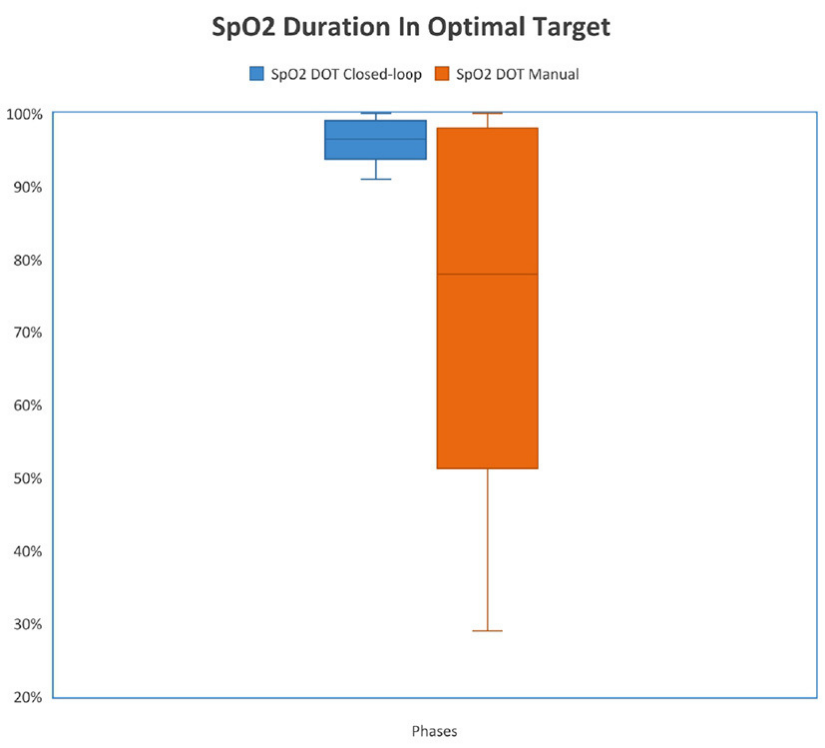
Comparison of duration in optimal target (DOT) SpO2 between study phases. {The median percentage of time spent in the optimal zone was 96.4% [93.6–98.7 (IQR)] when the FiO2 controller was activated and the median percentage of time spent in the optimal zone was 78.4% [51.4–98 (IQR)] when FiO2 was controlled manually (*p* < 0.001)}.

**Figure 4:**
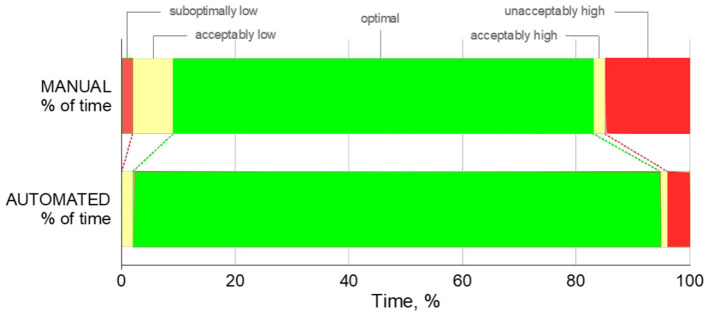
Percentage of time spent in unacceptably high/low, acceptably high/low, optimal SpO_2_ zones. For illustrative purposes, width of each bar represents the mean percentage of time spent.

Patients spent less time in the unacceptably low, sub-optimally low, acceptably low, and sub-optimally high zones while the FiO_2_ controller was activated with *p*-values 0.032, 0.008, 0.004 and 0.001, respectively. There was no significant difference at the time spent in the acceptably high zone (*p* = 0.151). A comparison of the percentage of time spent in optimal SpO_2_ ranges is shown in Figures 3, 4.

### Number of adjustments made to the FiO_2_ controller

Another secondary outcome was the number of adjustments made to the FiO_2_ controller. In the phase of closed-loop FiO_2_ control, the median number of adjustments was 52 [11.8–67 (IQR)], while the number of FiO_2_ adjustments during the manual phase was 1 [0–2 (IQR)].

### Subgroup analysis

For the normal PEEP group (<10 cm H2O), the median SpO_2_ in the closed-loop phase was 94.7% [93.6–95.4 (IQR)], whereas the same value for the manual phase was 95.6 % [93.2–96.4 (IQR)] (*p* = 0.229). For the higher PEEP group (≥10 cm H2O), the median SpO_2_ in the closed-loop phase was 89.4% [87.6–90.9 (IQR)], whereas the same value for the manual phase was 90 % [88.1–92.1 (IQR)] (*p* = 0.688). For the closed-loop phase the median OI ratio was 3.1 [2.4–6 (IQR)], the median FiO2 was 31.4% [25.1–44.1 (IQR)], and the median therapeutic O2 usage was 0.3 L/min [0.1–0.7 (IQR)], whereas the same values for the manual phase was 3.5 [2.6–6.6 (IQR)], 36.5% [30–49.1 (IQR)] and 0.5 L/min [0.2–0.9 (IQR)], respectively.

Pediatric ARDS subgroup with closed-loop FiO_2_ control, patients spent 95.9% [81.6–98.8 (IQR)] of their time in the optimal range compared to 78% [49.5–94.8 (IQR)] of their time in the optimal range when FiO_2_ was controlled manually. Also, they spent less time in the unacceptably low, sub-optimally low, acceptably low, and sub-optimally high zones while the FiO_2_ controller was activated, too. In the phase of closed-loop FiO_2_ control, the median number of adjustments for the PARDS subgroup was 57 [47–69 (IQR)], while the number of FiO_2_ adjustments during the manual phase was 1 [0–2 (IQR)].

## Discussion

In this randomized crossover clinical trial, we compared the percentage of time spent in the target SpO_2_ range during closed-loop FiO_2_ control vs. during manual control of FiO_2_ by the physician in pediatric intensive care patients ventilated in ASV mode. This rule based proportional integral closed-loop FiO2 controller is commercially available since 2011. The groups with a target oxygenation range of 93% to 97% when set PEEP was below 10 cm H2O and 88% to 92% when set PEEP was equal or above 10 cmH2O, may represent the vast majority of mechanically ventilated patients in pediatric intensive care. The median time spent in the target SpO_2_ range was 96.1% [93.7–98.6 (IQR)] during the closed-loop phase, whereas the same value was 78.4% [51.3–94.8 (IQR)] in the manual phase. This result was consistent with similar studies performed previously in preterm infants (4, 12–16). We can conclude that the automated FiO_2_ controller performs better and is more effective than manual setting of FiO_2_ in terms of maintaining patients in the optimal SpO_2_ range. The median FiO_2_ in the closed-loop phase was 31.4%, whereas the same value during the manual phase was 36.5% (*p* < 0.001). The median oxygenation index (OI) in the closed-loop phase was 3.1 [2.4–6 (IQR)], whereas the same value during the manual phase was 3.5 [2.6–6.6 (IQR)]. We also calculated the therapeutic O_2_ usage, with the median O_2_ usage being 0.5 L/min [0.2–0.9 (IQR)] in the manual phase, decreasing to 0.3 L/min [0.1–0.7 (IQR)] in the automated phase. This decrease in FiO_2_ use and OI may represent a more efficient use of therapeutic oxygen. A similar decrease was also noted by Bourassa et al. with their automated FiO_2_ titration device (17). Considering the surge in demand for mechanical ventilation and the possible scarcity of oxygen gas supply for these patients during pandemics, closed-loop FiO_2_ control may contribute to a more sparing use of this therapeutic agent (18–20). Friedman et al. aimed to characterize mechanical ventilation patterns in children receiving VV-ECMO and explore whether such practices are correlated to clinical outcomes (21). They found that after adjusting for sickness severity, FiO2 remained the only variable that could be modified. They also found that a 10% rise in FiO2 may be associated for 38% increase of the mortality of aforementioned group (21). The median FiO_2_ percentage during the automated phase was 31.4 [25.1–44.1 (IQR)], which was less than the median FiO_2_ during the manual phase at 36.5 [30–49.1 (IQR)]. Based on Friedman's study, it is possible that closed-loop FiO_2_ control may lower mortality in this population. The median number of manual adjustments required for patients in the manual phase of the study period was 1 [0–2 (IQR)], which means that the clinician may have to change the FiO_2_ setting at least 12 times per day. Bearing in mind the need during a pandemic for isolation measures and for donning personal protective equipment before entering an isolation room each time an adjustment is made, there may have been too little appreciation of this advantage with a closed-loop system prior to the pandemic.

Due to the small number of patients enrolled in the study, PARDS subgroups cannot be statistically analyzed. Furthermore, the automated FiO_2_ controller can be used only in ASV mode, which does not allow us to analyze the performance of the oxygen controller in other modes and patients below 7 kg of IBW. However, the ASV 1.1 algorithm was recently found to be safe in pediatric patients in terms of using less driving pressure (22). Another limitation was the unblinded bedside clinicians, who were aware that the closed-loop FiO_2_ controller was active. More stringent manual titration policy in the control group may have benefited the control group. However, we tried to compare closed- loop FiO_2_ control vs. standard care applied at the study site. Lastly, 2-h comparisons may not represent the whole course of a real clinical condition; however, due to the need for stable and similar oxygen demand we had to change patients over after 2 h.

## Conclusion

In this randomized crossover trial in pediatric patients with different lung conditions, the percentage of time spent in the target SpO_2_ range in ASV 1.1 with closed-loop FiO_2_ control was greater compared to the time spent in the target SpO_2_ zone in ASV 1.1 with manual FiO_2_ control. The use of closed-loop FiO_2_ control with ASV 1.1 may therefore contribute to maintaining ventilation in the target range of SpO_2_ in invasively ventilated pediatric patients.

## Data availability statement

The datasets generated and/or analyzed during the current study are not publicly available due to an IRB decision which was made in the interest of ensuring patient confidentiality but are available from the corresponding author on reasonable request. Requests to access these datasets should be directed to GC, drgokhanceylan@gmail.com.

## Ethics statement

The studies involving human participants were reviewed and approved by Institutional Review Board, Dr. Behcet Uz Children's Research and Training Hospital, Izmir, Turkey. Written informed consent to participate in this study was provided by the participants' legal guardian/next of kin.

## Author contributions

ES, GC, ST, PH, MC, OS, UK, GA, and HA conceived and designed the study. ES, GC, ST, PH, MC, MS, FS, OS, and UK acquired and analyzed. GC, FS, GA, DN, MS, and HA interpreted the data. GA, GC, and HA did the statistical analysis. ES, GC, DN, MS, and HA drafted the manuscript. OS, GC, ST, PH, MC, ES, UK, and HA had full access to all of the data. All authors critically revised the manuscript for important intellectual content, responsible for the final decision to submit for publication, and have seen and approved the manuscript.

## Funding

The participating centers received only material support from Hamilton Medical AG. They received ventilators for the duration of this study.

## Conflict of interest

Authors GC, DN, and MS report conflicts of interest, they are both employed by Hamilton Medical AG in the Department of Medical Research. The remaining authors declare that the research was conducted in the absence of any commercial or financial relationships that could be construed as a potential conflict of interest. The handling editor DB declared a past co-authorship with the author MS.

## Publisher's note

All claims expressed in this article are solely those of the authors and do not necessarily represent those of their affiliated organizations, or those of the publisher, the editors and the reviewers. Any product that may be evaluated in this article, or claim that may be made by its manufacturer, is not guaranteed or endorsed by the publisher.
