# Supplementary material for: Automated closed–loop FiO2 titration increases the percentage of time spent in optimal zones of oxygen saturation in pediatric patients–A randomized crossover clinical trial
Source: Front Med (Lausanne). 2022 Aug 25;9:969218. doi: 10.3389/fmed.2022.969218 (PMC9452913; doi:10.3389/fmed.2022.969218)
Supplement: Supplementary file 1 [file Table_1.DOCX]

Supplementary Table 1, Closed-loop oxygen controller algorithm and manual oxygen titration protocol

| **Oxygenation management** | **Action** | **Takes place when** |
| --- | --- | --- |
| Increase  Oxygen stepwise | Increases Oxygen by 10% of current Oxygen value every **30 seconds** | Oxygen automatically managed  Increasing Oxygen support. |
| Decrease Oxygen stepwise | Decreases Oxygen by 5% of current Oxygen value every **60 seconds** | Oxygen automatically managed  Decreasing Oxygen support  **NOTE:** If a lower limit is specified, Oxygen will not go below the lower limit. |

INTELLiVENT-ASV oxygenation controller calculates the difference between the actual SpO_2_ measurement and the target SpO_2_ range set by the clinician in order to determine the treatment action. Depending on the difference between the measured and the target SpO_2_ range: When the patient’s SpO_2_ is within the target SpO_2_ range, oxygen is fine-tuned to get the measured SpO2 to the middle of the target range. When the patient’s SpO_2_ is on the right of the target SpO2 range (high SpO_2_), the treatment is decreased. When the patient’s SpO_2_ is on the left of the target SpO2 range (low SpO2), the treatment is increased. When the measured SpO_2_ falls below 85% to 88%, depending on the SpO_2_ target range, oxygen is immediately increased to 100% as a safety emergency response. A medium priority alarm “FiO_2_ set to 100% due to low SpO_2_” is generated.

During the manual FiO2 control phase, the doctors were aware of both the PEEP and the resulting SpO_2_ goal. For SpO_2_, 85% and 99% were chosen as the low and high alarm thresholds, respectively. When the patient exceeded these alarm thresholds, they immediately titrated the FiO_2_ manually by 5 to 20%, if possible. Additionally, they manually titrated FiO2 by 5 to 20%, if possible when the patient spent more than 10 minutes beyond the target range but still inside the high and low alert limits.

FiO_2_ limitations weren't included in either the closed-loop FiO_2_ controller or the manual titrations. They have wide discretion between FiO_2_ concentrations of 21% and 100%.
